# Amino Acids Biostimulants and Protein Hydrolysates in Agricultural Sciences

**DOI:** 10.3390/plants13020210

**Published:** 2024-01-11

**Authors:** Wenli Sun, Mohamad Hesam Shahrajabian, Yue Kuang, Na Wang

**Affiliations:** National Key Laboratory of Agricultural Microbiology, Biotechnology Research Institute, Chinese Academy of Agricultural Sciences, Beijing 100086, China; hesamshahrajabian@gmail.com (M.H.S.); apcolyptyo@163.com (Y.K.); WangNa@163.com (N.W.)

**Keywords:** amino acids, biostimulants, medicinal plants, phenols, protein hydrolysates

## Abstract

The effects of different types of biostimulants on crops include improving the visual quality of the final products, stimulating the immune systems of plants, inducing the biosynthesis of plant defensive biomolecules, removing heavy metals from contaminated soil, improving crop performance, reducing leaching, improving root development and seed germination, inducing tolerance to abiotic and biotic stressors, promoting crop establishment and increasing nutrient-use efficiency. Protein hydrolysates are mixtures of polypeptides and free amino acids resulting from enzymatic and chemical hydrolysis of agro-industrial protein by-products obtained from animal or plant origins, and they are able to alleviate environmental stress effects, improve growth, and promote crop productivity. Amino acids involve various advantages such as increased yield and yield components, increased nutrient assimilation and stress tolerance, and improved yield components and quality characteristics. They are generally achieved through chemical or enzymatic protein hydrolysis, with significant capabilities to influence the synthesis and activity of some enzymes, gene expression, and redox-homeostasis. Increased yield, yield components, and crop quality; improved and regulated oxidation-reduction process, photosynthesis, and physiological activities; decreased negative effects of toxic components; and improved anti-fungal activities of plants are just some of the more important benefits of the application of phenols and phenolic biostimulants. The aim of this manuscript is to survey the impacts of amino acids, different types of protein hydrolysates, phenols, and phenolic biostimulants on different plants by presenting case studies and successful paradigms in several horticultural and agricultural crops.

## 1. Introduction

Biostimulants are considered bioactive substances that are either inorganic or organic microorganisms that can increase crop performance when utilized in small quantities [1] as they can enhance both performance and growth as well as improve nutrient- and water-use efficiencies of different crops [2,3,4,5,6,7,8]. Amino acids have a dual function as building blocks for proteins and as providers of organic nitrogen, which can alleviate the negative impacts of drought and salt stress [9], and promote cell growth. They are vital in metabolite synthesis, growth, and development, and appropriate in plants because of their structure as protein units [10,11,12,13,14]. The positive effects of the foliar application of amino acids and biostimulants based on amino acids on both the qualitative and quantitative characteristics of *Foeniculum vulgare* Mill, *Coriandrum sativum* L., *Achillea millefolium* L., *Nigella sativa* L., *Ocimum basilicum* L., *Urtica pilulifera* L., *Mentha piperita*, *Calendula officinalis* L., and *Satureja hortensis* L. plants have been reported [11,12,13,14,15,16,17,18,19,20,21,22,23,24,25].

Amino acids used for the production of biostimulants are obtained from the chemical synthesis of plant proteins, such as algae, soybean, and corn, as well as from animal proteins by both chemical and enzymatic hydrolysis. Amino acids that have been used for foliar usage are the result of enzymatic hydrolysis from both animal and plant protein hydrolysates, and as it is very energy-consuming, foliar application is a common process in the agricultural industry. Protein hydrolysate is related to the product of the hydrolytic action of protease(s) on a pure protein sample, or a complicated proteinaceous sample [26,27], which is necessarily a mixture of peptides, free amino acids, and probably partially degraded proteins [28,29]. Protein hydrolysates and amino acids, which are also known as protein-based biostimulants, are usually readily available because of the abundance of raw materials and their affordable cost [30,31,32]. Protein-based biostimulants can usually be obtained from the hydrolysis of protein-rich agro-wastes, which includes chemical, thermal, and enzymatic processes, or a combination of them [33,34,35,36]. They are usually considered as a crude peptide mixture, and they are usually used as the initial raw material for bioactivity testing [37,38,39]. Fish protein hydrolysates are famous in different parts of the world for pharmaceutical, cosmetic, and nutritional usage [40,41,42].

Several studies have reported that biostimulants promote plant resilience, especially by improving antioxidant activity within the plant under negative environmental conditions [43,44]. It could behave directly on the plant through an adjustment of the nitrogen and carbon metabolisms and the plant hormonal profile, or indirectly through the microbiome [45]. Food-derived bioactive proteins have physiological impacts on major body systems, such as opioid agonists and opioid antagonists on the nervous system; anti-hyperlipidemic, anti-thrombotic, anti-oxidative, and anti-hypertensive effects on the cardiovascular system; cytomodulatory, immunomodulatory, and anti-microbial effects on the immune system; and mineral binding, anti-appetizing, and anti-microbial impacts on the gastrointestinal system [46,47,48,49,50]. Phenols have notable roles in plant development and growth [51,52,53], as they are products of secondary metabolic procedures and are generally converted from sugars via the pentose phosphate pathway, the manganiferous acid pathway, the glycolytic pathway, or the benzene-propane pathway [54,55,56]. Phenolic acids include a carboxylic acid group in addition to the basic phenolic structure and are categorized into hydroxybenzoic and hydroxycinnamic acids. Phenolic acids can be utilized to grow crops by soil and foliar application as well as seed treatment, but foliar utilization of phenolic acids is usually suggested. This research examines the scientific literature on biostimulants from 1990 to October 2022 by conducting a bibliometric analysis of the literature published on the Web of Science database, including more than one thousand articles. The goal of this review article is to survey the effects of different biostimulants, such as amino acids, protein hydrolysates, and phenols, by presenting case studies and successful paradigms in different agricultural and horticultural crops. The information provided is obtained from randomized control experiments, review articles, and analytical observations and studies that have been gathered from various literature sources such as PubMed, Science Direct, Scopus, and Google Scholar. The keywords used were the Latin and common names of different agricultural and horticultural species, amino acids, protein hydrolysates, phenols, phenolic biostimulants, and medicinal plants.

## 2. Amino Acids

Amino acids for the production of biostimulants are derived by chemical synthesis from plant proteins such as soybean, corn, algae, corn, etc., as well as from animal proteins by enzymatic and chemical hydrolysis [57,58,59,60,61,62]. Amino acids act as vital molecules with various physiological roles [63] and play an important function in seed germination [64,65], and under salinity stress, they can behave as osmolytes, which can promote stomatal opening control, transport regulation, enzyme activation, heavy metals detoxification, redox homeostasis maintenance, and gene expression [66,67,68,69,70]. Supplementing plants with environmentally friendly amino acid biostimulants can decrease the application of inorganic fertilizers [71,72].

**Figure 1:**
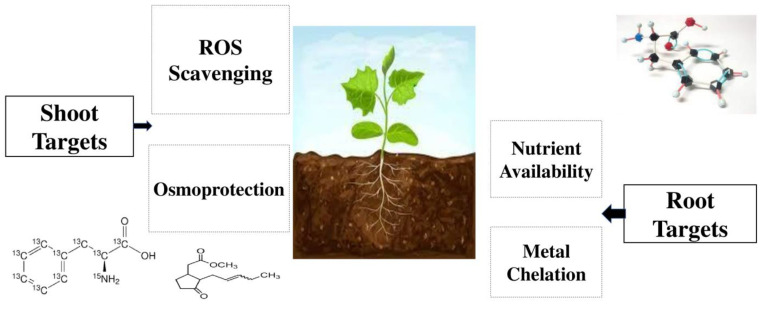
The most important mechanisms of amino acids biostimulants.

Amino acids are also important in the agriculture industry as chelates of metal ions and microelements chelated with amino acids from very small, electrically neutral molecules increase their transport and absorption within the plant [73,74,75]. Some of the most important products in the market which contain amino acids are Delfan Plus (Tradecorp, Madrid, Spain), Natural Crop SL (Natural Crop Poland Sp. Z o.o., Warsaw, Poland), Bosfoliar Activ (COMPO EXPERT, Munster, Germany), Amino Quelant Ca (Bioiberica, Barcelona, Spain), Tecamin Max, Tecamin Brix, Tecnokel Amino Mix, Terra-Sorb Foliar (Agritecno Fertilizants, Valencia, Spain), Agrocean B (Agrimer, Plouguerneau, France), Metalosate Calcium and Metalosate Fe (Albion Minerals, Layton, UT, USA) [76,77,78,79]. The usage of amino acids can increase co-enzyme formation and the photosynthesis procedure [80], and supports different plant organisms that may face environmental stresses [81]. It has been also reported that the exogenous utilization of amino acids can enhance nitrogen status, and the contents of mineral elements in plant tissues [82,83]. Depending on environmental conditions and plant species, plants reduce inorganic nitrogen to amino acids in roots, nodules, and leaves [84,85,86]. Many studies have reported the important and notable effects of the foliar application of concentrations with phenylalanine and tyrosine solutions on essential oil, the total amount of phenols, and their compositions in *Ocimum basilicum* L., *Melissa officinalis* L., and *Coleus blumei* L. plants [87,88,89]. Phenylalanine is an amino acid [90,91], and its foliar application can help mustard (*Brassica campestris* L.) plants overcome drought stress and increase total chlorophyll contents, shoot length, and biological yield [92]. Roman et al. [93] reported that foliar application of methyl jasmonate and phenylalanine can increase the content of volatile compounds in grapes, and Portu et al. [94] introduced it as an important management tool for boosting grape quality. The impacts of different amino acids on several experimental plants are shown in Table 1. The roles of different amino acids as biostimulants are shown in Table 2. The main mechanisms of amino acids biostimulants are shown in Figure 1.

## 3. Protein Hydrolysates

Protein hydrolysates, especially those that contain antioxidant peptides, are obtained from natural components, and many researchers and scholars consider them biostimulants because of their minimum side effects, easy absorption, low cost, high activity, and lower molecular weight [123,124,125,126,127,128,129,130]. Protein hydrolysates and peptides can be used as notable ingredients in the formulation of functional foods [131,132,133,134,135,136,137,138,139,140,141]. They can be used as foliar sprays or through drip irrigation systems, and the amino acids can be absorbed through both leaves and roots [142,143,144]. Their utilization can significantly affect nitrogen metabolism in plants, and boost productivity, particularly when applied as a seed pre-treatment [144]. For separating the amino acids in protein hydrolysates, a liquid chromatography process can be used [145,146]. Numerous methods have been considered to produce hydrolysates from fish and fish by-products such as thermal hydrolysis, autolysis, chemical hydrolysis, and enzymatic hydrolysis [146,147]. The basic procedures utilized following hydrolysis of protein are heat inactivation, which has a function in the inactivation of proteolytic enzymes; ultrafiltration, which is important in the removal of high molecular weight peptides and proteins; use of specific enzymes, which can reduce the content of specific amino acids; hydrolysis by exoproteases, which is active in hydrolysis and the reduction of bitterness; carob activation, which has a notable role in the reduction of bitterness; and absorption chromatography, which can decrease the content of aromatic amino acids. Microbial-based biostimulants such as Environoc 401^®^, Bioyield^®^, Rootshield Plus^+^ WP ^®^, Spectrum + Myco^®^, Select^®^, and Endomaxx^®^ inconsistently increased the quality of bell pepper (*Capsicum annuum* L.) in a greenhouse experiment [148]. Ghorbel-Bellaaj et al. [149] reported that five proteolytic enzymes, namely Alcalase^®^, trypsin, a crude enzyme extract from sardinelle (*Sardinella aurita*) viscera, and an enzyme preparation from *Aspergillus clavatus* ESA and *Bacillus licheniformis* NH1, which are protein hydrolysates, were obtained from shrimp via by-products processing, and they have revealed notable degrees of antioxidant activities, such as β-carotene bleaching, reducing power, and 1,1-diphenyl-2-picrylhydrazyl (DPPH)-scavenging activity assays, which can be a promising and helpful alternative for accessible commercial nitrogen sources from other origins. It can be a good source for microbial growth and protease production by *Saccharomyces cerevisiae*, *Escherichia coli*, *Bacillus subtilis* A26, and *Bacillus mojavensis* A21.

Some of the available plant biostimulants, their composition, and application strategies are C Fish, which contain peptides and amino acids that are used on vegetables and fruits to increase the plant’s resistance to insect pressure, disease and drought or heat stress which originates from white fish/mixed fish composition autolysates and hydrolysates in fruits and vegetables; Radifarm, which contains peptides, amino acids, betaines, saponins, vitamins, polysaccharides, and microelements, has been used to promote the formation of an extensive root system by speeding up the elongation of adventitious and lateral roots of vegetables and fruits; Megafol, which contains betaines, amino acids, auxin, vitamins, proteins, cytokine, and gibberellin, can improve the balance between vegetative productivity and development as well as plant resistance to stressors such as hail, weeding, root asphyxia, and frost; Biozyme, which includes plant hormones, algae extract, and chelated micronutrients, can boost nutrient uptake, photosynthesis, and the activity of chlorophyll of legumes, vegetables and fruits; BioRoot, which contains humates, plant and mineral-derived organic acids, enhances rooting ability, protein content, and chlorophyll of fruits and vegetables; Grow-plex SP, which contain humic acids, can increase soil bacteria, shoot and root growth, and zinc and iron uptake of vegetables and fruits; Ergonfil, which has cysteine, animal protein hydrolysates, keratin derivatives, and folic acid, can promote chlorophyll synthesis and indole acetic acid, increase chelation, and improve translocation in fruits and vegetables; Benefit, which contains nucleotides, amino acids, vitamins, free enzymatic proteins, can improve cell division and increase the number of cells per fruit [150,151,152,153]. Animal-derived gelatin, which has peptides and amino acids, can improve shoot dry weight and promote root nitrogen assimilation in broccoli, arugula, tomato, pepper, cucumber, and field corn [154]. There are notable reports and evidence that the application of non-structural and structural amino acids, such as histidine, proline, taurine, and glutamate, can provide protection to the plant from environmental stresses or play an important function in metabolic signaling by regulating nitrogen acquisition by the roots [155,156]. Amino acids can act as osmoprotectants, which stabilize membranes, enzymes, and proteins against denaturing caused by high salt components and non-physiological temperatures [157]; moreover, arginine has been proven to have an important function in nitrogen transport and storage in plants during biotic and abiotic stress conditions [158]. Amino acids can also reduce plant toxicity by heavy metals by acting as metal chelators [159,160]. Rouphael et al. [161] reported that the application of vegetal-protein hydrolysates based microgranules can increase carotenoids and total chlorophyll content. Protein hydrolysate has a positive influence on total root area and on root length, which can increase mineral-nutrient and water-use efficiency as well as promote plant productivity and resistance to harmful conditions [162,163,164]. It can also positively influence the leaf area and yield of horticultural plants and fruit trees [165,166]. The exogenous utilization of protein hydrolysate and isolated amino acids can promote plant antioxidant performance by improving the non-enzymatic and enzymatic antioxidant defense machinery of the cell [167]. The most important effects of different kinds of protein hydrolysates have been shown in Table 3.

## 4. Phenols and Phenolic Biostimulants

Phenols are a major type of antioxidant phytochemical, which have significant importance because of their free radical scavenging and biological characteristics [233,234,235,236]. Phenolic compounds are the most abundant secondary metabolites in many plants which are usually discovered in the cell walls of subepidermal and in the vacuoles of epidermal cells [237,238]. Endogenous phenolic components in plants have different functions, which can be used by plants to defend themselves against pathogens, herbivores, and weeds. They are implicated in seed germination and dormancy, appropriate as screens against damaging UV radiation, and act as pigments to attract seed dispersal agents and pollinators [239,240,241]. The function of phenolic acids as signaling molecules in plant-microbe symbioses has been reported in previous research [242]. Some of the most important phenolic compounds with bioprotectant activities are ferulic acid, curcumin, ellagic acid, catechol, gallic acid, coumarin, caffeic acid, catechin, quercetin, sinapic acid, rutin, resveratrol, salicylic acid, and syringic acid [243,244]. The accumulation of phenolic compounds and the subsequent production of quinones in turnip (*Brassica rapa* L.) may happen when plants are susceptible to Boron deficiency [245]. Phenolic compound concentration can be important in the biochemical pathway of toxigenic fungal species because of the induction of stress via sub-lethal contents and depletion of the phenolic compounds [246]. Phenolics have meaningful functions in plant development, especially in pigment and lignin biosynthesis as well as considerable roles in plant protection against stress. It has been reported the correlation between antifungal activity and total phenolics of plants [247] and the accumulation of amino acids and phenolics may boost tolerance to both copper and cobalt stress in barley [248]. Silva et al. [249] reported that tyrosol, which is a phenolic compound from olive oil and several endophytic fungi such as *Phomopsis* sp., can be used as an important biostimulant in soybean seed treatment, which can alter soybean plant metabolism without meaningful impacts on crop yield. Masondo et al. [250] reported that two phenolic biostimulants, namely eckol and phloroglucinol, isolated from brown algae *Ecklonia maxima* can have a significant effect on the phytochemical and growth of *Eucomis autumnalis*. While the phenolic acid metabolism in *Kandelia obovata* may decrease the negative impacts of cadmium and zinc [251], it has been reported that the phenolic compounds of leave extracts of *Calligonum arich* L. are effectual against pathogenic bacteria [252], and the phenolic compounds of apricot branches have shown antifungal activity against *Monilinia laxa* growth [253,254,255]. One of the notable impacts of phenolics is to improve the resistance of *Nicotiana langsdorffii* to Cr(VI) [256,257,258,259].

## 5. Conclusions and Future Prospects

The innovative agronomic tools of agriculture are biostimulants, which are composed of inorganic and organic substances that consist of several microorganisms and substances. Biostimulants can do various agronomic functions such as increasing the growth and development of plants during their entire life cycle; promoting the resistance of plants to abiotic stresses such as cold, heat, and lack of water; improving soil fertility, especially increasing the development of soil microorganisms; promoting the use efficiency of nutrients by plants; and finally, increasing yield and crop quality. They can also be used as the best alternative for chemical fertilizers and are the best strategy for promoting organic agriculture. Amino acids are appropriate candidates to boost stress tolerance through metal chelation, nutrient availability, osmo-protection, and reactive oxygen species (ROS), which can notably affect the synthesis and stimulation of gene expression and some enzymes. Amino acids are organic components, which contain amine and carboxyl C(=O)OH) functional groups together with a side chain (R group). They can promote and stimulate the process of protein synthesis and photosynthesis; promote nutrient assimilation, translocation, and utilization; and strengthen plant growth and yield formation. Protein hydrolysates are manufactured from plant-derived protein sources using partial thermal hydrolysis, chemical hydrolysis, and enzymatic hydrolysis. Different sources of protein hydrolysates on the basis of protein sources are animal origin, leather by-products, blood meal, fish by-products, chicken feathers, casein, plant origin, legume seeds, alfalfa hay, and vegetable by-products. The positive impacts of the utilization of amino acids have been discovered; however, there is not enough knowledge about the effects of each amino acid on both the physiological and metabolic processes of plants. A better understanding of biostimulants, such as amino acids, protein hydrolysates, phenols, and phenolic biostimulants, while considering their various effects on different functions of crops, namely crop yield and yield components, growth promotion, and nutrient availability, may help agricultural scientists and farmers to better understanding and utilization of them.

## Figures and Tables

**Table 1:** The effects of amino acids on different plants.

Plant	Plant Family	Key Point	Reference
Chickpea (*Cicer arietinum* L.)	Fabaceae	The combined application of amino acids of commercial compounds with proline + valine, and proline + alanine can reduce the negative impacts of drought stress on chickpeas.	[95]
Cowpea (*Vigna unguiculata*)	Fabaceae	The foliar application of amino acid liquid fertilizer and liquid biological fertilizer can enhance crop yield.	[96]
Grapevine (*Vitis vinifera* L.)	Vitaceae	A biostimulant that contains amino acids can enhance the growth of the microbial community on berry skin.	[97]
Lettuce (*Lactuca sativa* L.)	Asteraceae	The foliar utilization of amino acids biostimulants (Perfectose^TM^, liquid) can improve the nutritive value and yield of lettuce.	[98]
		An amino-acid-based Phytostim^®^ biostimulant can improve growth and yield attributes.	[99]
		The biostimulant Codasil^®,^ which is composed of amino acids, can improve lettuce physiology and growth, and enhance the crop resistance to water stress.	[100]
		The application of proline and methionine increased proteinogenic amino acid expression.	[101]
		Terra Sorb^®^ radicular and Terramin^®^ Pro, which contain high amino-acid content, are useful biostimulants for plant development in nitrogen-limiting areas.	[102]
Mint (*Mentha* × *Piperita* L.)	Lamiaceae	The application of phenylalanine at 100 mg L^−1^ concentration enhanced the essential oil.	[103]
Moldavian balm (*Dracocephalum moldavica* L.)	Lamiaceae	Leaf spraying of biostimulants based on amino acids can notably mitigate the adverse impact of salinity stress on the growth and physiological growth of plants.	[104]
Strawberry (*Fragaria* × *ananassa*)	Rosaceae	The combined application of humic acids and amino acids can improve strawberry nutritional traits such as phenolic compounds, and commercial characteristics such as external color and firmness.	[105]
Sunflower (*Helianthus annuus* L.)	Asteraceae	Plant biostimulant Amino Expert^®^ Impuls can increase sunflower plant height, head diameter, seed yield, seed oil, and absolute seed mass.	[106]
Tomato (*Solanum lycopersicum* L.)	Solanaceae	The combined application of amino acids and humic acids could significantly influence yield in conventional nutrition.	[107]
		The combined application of amino acids and humic acids could positively improve total antioxidant capacity, total flavonoid content, and total phenol content.	[108]
		Biostimulants that contain amino acids could increase the accumulation of plant biomass as well as improve the tolerance of plants to water deficit.	[109]
		The application of amino acids can induce a higher accumulation of total soluble sugars.	[110]
Olive (*Olea europaea* L.)	Oleaceae	The complex of natural amino acids, such as hydroxyproline, proline, and glycine, can induce higher stomatal conductance and leaf photosynthetic rates.	[111]
		The combined application of amino acids, fulvic acid, and humic acid can significantly increase the quality and the oil content of olives.	[111]
Peanut (*Arachis hypogaea* L.)	Fabaceae	Foliar utilization of 100 mg/L aspartic acid can increase seed and oil yield.	[112]
Pepper (*Capsicum annuum* L.)	Solanaceae	Actium^®^, provided by Grupo Agrotecnologia (Alicante, Spain), contains amino acids that could enhance carotenoids, total monosaccharides, and phenylalanine in plants.	[113]
		The application of biostimulants that contain amino acids can boost the activity of important enzymes such as peroxidase, phenylalanine ammonia lyase, and capsaicin synthase.	[114]
Rice (*Oryza sativa* L.)	Poaceae	Zinc-enriched amino acids (Zn-AAC) increased salt-stressed rice yield, chlorophyll content, and quality of rice.	[115]
		The combined application of potassic fertilizer with amino acids can improve both the yield and quality of rice.	[116]
Soybean (*Glycine max* L.)	Fabaceae	Amino acid application can increase plant height, the number of seeds and pods, flavonoid content, and phenolic content.	[117]
		The application of phenylalanine and cysteine could enhance the production of soybean plants by at least 21%.	[118]
Spinach (*Spinacia oleracea* L.)	Amaranthaceae	The application of different amino acid treatments such as tyrosine, methionine, proline, and phenylalanine could increase dry and fresh weight, shoot length, root length, leaf area, and final yield.	[119]
Weeping alkaligrass (*Puccinellia distans*)	Poaceae	Two biostimulants, namely Bonamid^®^ at 2 g/L, and Algabon^®^ at 0.5 g/L, which contained amino acids could positively increase K^+^ content, chlorophyll content, K^+^/Na^+^ ratio, leaf relative water content, and biomass as well as reduce the adverse effects of NaCl-caused stress in vacuoles.	[120]
Winter wheat (*Triticum aestivum* L.)	Poaceae	INTERMAG Co. (Olkusz, Poland)—AminoHort and AminoPrim, containing 20% and 15% amino acids at 1.25 L/ha and 1.0 L/ha, could significantly increase nutrient contents such as molybdenum, calcium, sodium, and copper in grains.	[121]
		The application of amino acids together with yeast extract can significantly boost physiological yield and traits.	[122]

**Table 2:** The roles of different amino acids as biostimulants.

Amino Acids	Function	References
Deflan Plus	Increase co-enzyme formation; improve photosynthesis procedure; increase resistant of plants to environmental stresses	[76,77,78,79,80,81]
Natural Crop SL	Increase co-enzyme formation; improve photosynthesis procedure; increase resistant of plants to environmental stresses	[76,77,78,79,80,81]
Tecamin Max	Increase co-enzyme formation; improve photosynthesis procedure; increase resistant of plants to environmental stresses	[76,77,78,79,80,81]
Tecamin Brix	Increase co-enzyme formation; improve photosynthesis procedure; increase resistant of plants to environmental stresses	[76,77,78,79,80,81]
Agrocean B	Increase co-enzyme formation; improve photosynthesis procedure; increase resistant of plants to environmental stresses	[76,77,78,79,80,81]
Metalosate Calcium	Increase co-enzyme formation; improve photosynthesis procedure; increase resistant of plants to environmental stresses	[76,77,78,79,80,81]
Metalosate Fe	Increase co-enzyme formation; improve photosynthesis procedure; increase resistant of plants to environmental stresses	[76,77,78,79,80,81]
Phenylalanine and tyrosine solutions	Improve essential oil, and increase the total amount of phenols	[87,88,89]
Phenylalanine	An important amino acid that can enhance shoot length, biological yield, and total chlorophyll contents	[90,91,92,93]
Methyl jasmonate	Enhance the content of volatile components	[93,94]
Proline, Valine, Alanine	They can reduce the adverse effects of drought stress	[95]
Perfectose^TM^	It can increase the yield and nutritive value of plants	[98]
Phytostim^®^	It can increase final yield and growth	[99]
Codasil^®^	It can increase resistance to drought stress	[100]
Sorb^®^, Radicular, Terramin^®^	They are appropriate to improve the yield of plants in nitrogen-limiting regions	[102]
Amino Expert^®^	It may increase yield and yield components	[106]
Actium^®^	It can increase carotenoids and quality parameters	[113]
Bonamid^®^, Algabon^®^	They can increase chlorophyll content, biomass, and leaf relative water content	[120]
INTERMAG Co. (Olkusz, Poland)—AminoHort, and AminoPrim	It can improve mineral components in plants	[121]

**Table 3:** The impacts of different protein hydrolysates on various plants.

Plant	Plant Family	Protein Hydrolysate	Key Point	Reference
Apple (*Malus domestica*)	Rosaceae	Alfalfa protein hydrolysate	It can improve sensorial characteristics and fruit quality. Promote nutraceutical value, and decrease post-harvest disease.	[168]
Banana (*Musa acuminata*)	Musaceae	Chicken feathers hydrolysate	Promote chlorophyll content and increase photosynthetic. Increase fruit yield, filling, and set as well as antioxidants and decrease time to flowering	[169]
Basil (*Ocimum basilicum*)	Lamiaceae	Protein hydrolysate	It can decrease nitrate leaf content, and enhance basil resilience.	[170]
Castor (*Ricinus communis*)	Euphorbiaceae	Soybean protein hydrolysate (SPH)	It could lead to a significant increase in castor husks and final yield.	[171]
Celery (*Apium graveolens* L.)	Apiaceae	Protein hydrolysates	It can boost the total phenolic content in plants.	[172]
Chickpea (*Cicer arietinum* L.)	Fabaceae	Chicken feathers hydrolysate	Increases secondary roots and biomass production, and reveals phytohormone-like activities.	[173]
Common bean (*Phaseolus vulgaris* L.)	Fabaceae	Pumpkin seed protein hydrolysate	Application of 2000 μL L^−1^ to obtain appropriate yield and growth of plants under salt stress.	[174]
Coriander (*Coriandrum sativum*)	Apiaceae	Commercial amino acid preparation	It has glycine. which can improve the growth of shoots and leaves and increase the micronutrient content of leaves.	[175]
Florist’s daisy (*Chrysanthemum morifolium*)	Asteraceae	Two plant protein hydrolysates (Trainer^®^, and Vegamin©), and one animal protein hydrolysate (Hicure^®^)	Plant protein hydrolysates could decrease nitrate concentration in flowers and leaves. Animal protein hydrolysate caused a faster duration of flower stems to wilt stage.	[176]
Grape tomatoes (*Solanum lycopersicum* var. cerasiforme)	Solanaceae	Fish-derived protein hydrolysates	Application of fish-derived protein hydrolysates could reduce the negative impacts of drought, and improve total plant biomass yield, leaf dry weight, and fruit number.	[177]
Grapevine (*Vitis vinifera* L.)	Vitaceae	Protein hydrolysates Trainer and Stimtide	Both of them induced alterations in leaf metabolome and proteome, which can delay physiological maturity and keep higher acidity.	[178]
		Animal- and plant-derived protein hydrolysates, namely lupin, soybean, and casein	It can increase fruit parameters and alleviate the adverse effects of water stress.	[179]
Hemp (*Cannabis sativa* L.)	Cannabaceae	A commercial legume-derived protein hydrolysate	It can increase seed yield and improve fiber production.	[180]
		Fish hydrolysate, *Aloe vera*, and Kelp	It can increase branching, root growth, and propagation effectiveness as well as improve potassium and phosphorous uptake.	[181]
Hibiscus (*Hibiscus moscheutos* L. subsp. *palustris*)	Malvaceae	Protein hydrolysates from biowaste as biostimulants	It could improve leaf gaseous exchanges, biometric parameters, nitrogen-use efficiency, and biomass accumulation.	[182]
Kiwifruit (*Actinidia deliciosa*)	Actinidiaceae	Gelatin hydrolysate	Increase root and shoot biomass. Boost metabolism and assimilation of nitrogen.	[183]
Lettuce *(Lactuca sativa* L.)		Fish-derived protein hydrolysate	It contains amino acids and peptides, which can improve root biomass and leaf number and enhance photosynthetic rate and chlorophyll content.	[184]
Lettuce (*Lactuca sativa* L.)	Asteraceae	Commercial amino acids preparation	It has glutamine and glycine, which can enhance vitamin C content, leaf chlorophyll, and yield.	[185]
		Protein hydrolysates	Application of Molybdenum dosage together with protein hydrolysates can increase yield, nutritional, morphology, and functional features.	[186]
		Soy protein hydrolysate	Application of 0.01 mg/mL protein hydrolysate can promote lettuce weight and length.	[187]
		A *Graminaceae*-derived protein hydrolysate	It can improve the growth and yield of plants and improve the resistance of plants under mild salinity conditions.	[188]
		Plant-derived protein hydrolysates	It can improve root dry weight and dry biomass and increase fresh yield.	[189]
		Protein hydrolysate derived from pig blood	Its application can improve anthocyanins and flavonoids as well as root and shoot fresh weight.	[190]
Maize (*Zea mays* L.)	Poaceae	Soybean protein hydrolysate (SPH)	Application of fertilizer with SPH can increase one thousand grain weight, the grain number per ear, and total yield.	[188,189]
		A solid biostimulant (AA309) derived through thermobaric hydrolysis applied on trimmings and shavings of bovine hides tanned with wet-blue technology	It can improve the yield of crops. It can influence plant physiology because of changes they can induce in plant-associated microbes^,^ composition and activity.	[190]
		Kaishi, a protein hydrolysate-based biostimulant	It can promote root and shoot growth and increase lipid peroxidation.	[191]
		Meat flour protein hydrolysate	It contains amino acids and peptides, which can improve leaf and root biomass and promote effective nutrient utilization by plants.	[192]
		Chicken feather hydrolysates	Amino acids and peptides can increase macronutrient and micronutrient concentrations of leaves and grain protein content.	[193]
		A novel biostimulant (APR^®^, ILSA S.p.A., Arzigano VI, Italy)	It can influence shoot and root growth and improve the resistance of plants to various stresses.	[194]
		A commercial collagen-derived protein hydrolysate	It can stimulate lateral root growth and final yield.	[195]
Melon (*Cucumis melo* L.)	Cucurbitaceae	Fish protein hydrolysates	It can promote the activity of sucrose phosphate synthase. It can improve fructose and glucose contents by increasing the activity of acid invertase. It can boost the synthesis direction of sucrose synthase.	[196]
Oregano *(Origanum vulgare* L.)	Lamiaceae	Fish protein hydrolysates at 1000 mg/L	It can prevent vitrification in oregano shoot clones regenerated from axillary bud explants. Fish protein hydrolysate-treated shoots can decrease elongation and induce higher chlorophyll content.	[197]
Passion fruit (*Passiflora Edulis*)	Passifloraceae	Commercial preparation of peptides and amino acids	It has peptides and amino acids, which can increase the photosynthetic process in plants and increase transplanting success.	[198]
Pea (*Pisum sativum* L.)	Fabaceae	Papain and pepsin-hydrolyzed whey protein	Application of 2000 mg/L of biostimulant can increase pod length, pod growth, and the number of seeds per pod.	[199]
Peppermint (*Mentha* × *piperita* L.)	Lamiaceae	Amino16^®^, a commercial protein hydrolysate	It could not impact dry or fresh weight; however, it decreased plant height. It promoted total soluble phenol and total antioxidant capacity.	[200]
Persimmon (*Diospyros kaki*)	Ebenaceae	Protein hydrolysate	Increases the biosynthesis of salt stress response proteins	[201]
Rapeseed (*Brassica napus* subsp. *napus*)	Brassicaceae	Soybean protein hydrolysate (SPH)	It improved yield and promote the growth of plants.	[202]
Rice (*Oryza sativa* L.)	Poaceae	Soybean protein hydrolysates	It can decrease long- and short-term retrogradation of gelatinized rice starch.	[203]
Sea grape (*Coccoloba uvifera* L.)	Polygonaceae	Jackfruit (*Artocarpus heteropyllus* L.) leaf protein hydrolysates	It has shown emulsifying properties, and it could be used as an alternative to conventional emulsifiers.	[204]
Snapdragon (*Antirrhinum majus* L.)	Plantaginaceae	Protein hydrolysates	The combined application of protein hydrolysates, humic acids, and seaweed extracts could increase the number of leaves and improve the performance of ornamental plants.	[205]
Soybean (*Glycine max* L.)	Fabaceae	Protein hydrolysates	It can improve the final yield of plants.	[206]
Spearmint (*Mentha spicata* L.)	Lamiaceae	Amino16^®^, a commercial protein hydrolysate	It could increase the quality of spearmint without negative impacts on crop yield.	[207]
Spinach (*Spinacia oleracea* L.)	Amaranthaceae	*Xcell Boost*, a mixture of fish protein hydrolysates and kelp extract	It is highly beneficial for promoting the tolerance of spinach to water shortage stress.	[208]
		Trainer^®^, a plant-derived protein hydrolysates	It can increase total amino acid content, but reduce polyphenol content and increase final yield.	[209]
Sweet basil (*Ocimum basilicum* L.)	Lamiaceae	Animal-derived protein hydrolysate	It can decrease plant growth, photosynthetic performance, and yield.	[210]
Sugar beet (*Beta vulgaris*)	Amaranthaceae	Protein-based biostimulants (PBBs)	The application of 2 g/kg soil PBBs increased protein-related characteristics in samples and induced higher photosynthesis, growth, and quality of plants.	[211]
		Hydrolyzed wheat gluten and potato protein	It can enhance final yield and plant growth.	[212]
Sweet cherry (*Prunus avium* L.)	Rosaceae	An organic fertilizer (Defender Ca; Kenya Biologics Ltd., Runyenjes, Kenya)	It can improve fruit yield, soluble solids content, and calcium concentration in fruits.	[213]
Sweet pepper (*Capsicum annuum* L.)	Solanaceae	Organic fertilizer based on hydrolyzed proteins	It could improve the performance in nitrogen uptake, increase resistance to tolerance, and mitigate the negative impacts of toxic elements.	[214]
Sweet potato (*Ipomoea batatas* L.)	Convolvulaceae	Whey protein hydrolysates (WPH)	Foliar application of WPH at 0.10 and 0.20% could improve uptake of K, P, and N by shoots, shoot dry weight per plant, final yield, marketable yield, and total yield.	[215]
Tea (*Camellia sinensis*)	Theaceae	Chicken feather protein hydrolysate	It can be applied as a growth booster for gaining higher yields.	[216]
Tomato (*Solanum lycopersicum* L.)	Solanaceae	CycoFlow, Agriges, BN, Italy, a novel protein hydrolysate-based biostimulant	It can induce better pollen viability and water status as well as improve antioxidant contents in fruits and leaves.	[217]
		Soy protein hydrolysates (SPH13 and SPH18 at 10 g L^−1^)	It can notably improve plant resistance to foliar inoculation with *Pseudomonas syringae* pv. tomato DC3000.	[218]
		Protein hydrolysates	Its usage can stimulate plant growth.	[219]
		Protein hydrolysates	Its application could enhance fruit antioxidants such as ascorbic acid levels, polyphenols, and lycopene.	[220]
		Plant-derived protein hydrolysates	It can enhance nitrogen use and uptake as well as tomato yield.	[221]
		Protein hydrolysates	It can be considered an important biostimulant to improve plant resilience to abiotic stresses.	[222]
		*Arthrospira platensis* protein hydrolyzate	Its application as 68.9 mg mL^−^ free amino acids can improve plant yield and growth.	[223]
		The pig blood-derived protein hydrolysate	It can increase salt tolerance in tomatoes and improve photosynthetic efficiency, chlorophyll levels, and plant growth.	[224]
		Pig blood-derived protein hydrolysate	It can increase yield and mitigate the negative impacts of drought stress by regulating chloroplast ultrastructure, antioxidant systems, stomatal aperture, and osmotic changes.	[225]
		Legume-derived protein hydrolysate	Its application at 5.0 mL L^−1^ improved mineral composition, total soluble solids, and antioxidant activities.	[226]
		An enzymatically hydrolyzed animal protein-based biostimulant (Pepton)	It can show a positive impact, increasing the lateral and primary growth of tomato plants.	[227]
Wall rocket (*Diplotaxis tenuifolia* (L.) DC.)	Brassicaceae	Legume-derived protein hydrolysates and *Trichoderma harzianum* T22; Protein hydrolysates + *Trichoderma harzianum* T22	They can boost the hydrophilic and lipophilic antioxidant activity.	[228]
Wheat (*Triticum aestivum* L.)	Poaceae	AGROMOREE, a biostimulant based on a protein hydrolysate rainbow trout (*Oncorhynchus mykiss*)	It can increase gluten content, seed protein, and final productivity, and reduce the use of nitrogen fertilizers.	[229]
		Protein hydrolysate	It can improve wheat grain seed germination and improve final production.	[230]
		Papain-produced whey protein hydrolysates	It can improve spike number, flag leaf area, and grain yield.	[231]
White mustard *(Sinapis alba* L.)	Brassicaceae	Protein hydrolysate (Hemozym)	It can significantly increase the physicochemical properties and microbial activity of the soil.	[232]
